# Epigenetic Repression of *RARRES1* Is Mediated by Methylation of a Proximal Promoter and a Loss of CTCF Binding

**DOI:** 10.1371/journal.pone.0036891

**Published:** 2012-05-17

**Authors:** Zhengang Peng, Rulong Shen, Ying-Wei Li, Kun-Yu Teng, Charles L. Shapiro, Huey-Jen L. Lin

**Affiliations:** 1 Division of Medical Technology, School of Allied Medical Professions, the Ohio State University Medical Center, Columbus, Ohio, United States of America; 2 Molecular Biology and Cancer Genetics Program, Comprehensive Cancer Center, the Ohio State University Medical Center, Columbus, Ohio, United States of America; 3 Department of Pathology, the Ohio State University Medical Center, Columbus, Ohio, United States of America; 4 Department of Medical Oncology, the Ohio State University Medical Center, Columbus, Ohio, United States of America; 5 Department of Medical Technology, University of Delaware, Newark, Delaware, United States of America; Florida State University, United States of America

## Abstract

**Background:**

The *cis*-acting promoter element responsible for epigenetic silencing of *retinoic acid receptor responder 1* (*RARRES1*) by methylation is unclear. Likewise, how aberrant methylation interplays effectors and thus affects breast neoplastic features remains largely unknown.

**Methodology/Principal Findings:**

We first compared methylation occurring at the sequences (−664∼+420) flanking the *RARRES1* promoter in primary breast carcinomas to that in adjacent benign tissues. Surprisingly, tumor cores displayed significantly elevated methylation occurring solely at the upstream region (−664∼−86), while the downstream element (−85∼+420) proximal to the transcriptional start site (+1) remained largely unchanged. Yet, hypermethylation at the former did not result in appreciable silencing effect. In contrast, the proximal sequence displayed full promoter activity and methylation of which remarkably silenced *RARRES1* transcription. This phenomenon was recapitulated in breast cancer cell lines, in which methylation at the proximal region strikingly coincided with downregulation. We also discovered that CTCF occupancy was enriched at the unmethylayed promoter bound with transcription-active histone markings. Furthermore, knocking-down *CTCF* expression hampered RARRES1 expression, suggesting CTCF positively regulated *RARRES1* transcription presumably by binding to unmethylated promoter poised at transcription-ready state. Moreover, RARRES1 restoration not only impeded cell invasion but also promoted death induced by chemotherapeutic agents, denoting its tumor suppressive effect. Its role of attenuating invasion agreed with data generated from clinical specimens revealing that *RARRES1* was generally downregulated in metastatic lymph nodes compared to the tumor cores.

**Conclusion/Significance:**

This report delineated silencing of *RARRES1* by hypermethylation is occurring at a proximal promoter element and is associated with a loss of binding to CTCF, an activator for RARRES1 expression. We also revealed the tumor suppressive roles exerted by RARRES1 in part by promoting breast epithelial cell death and by impeding cell invasion that is an important property for metastatic spread.

## Introduction

Retinoic acid receptor responder (Tazarotene-induced gene) 1 (*RARRES1,* alias *TIG1*), initially identified as a downstream target of retinoic acid signaling [1], was demonstrated to be inactivated in primary tumors and cell lines of human cancers. Other than inhibiting tumorigenesis and hampering invasive properties of prostate cancer [2], increasing lines of evidence have indicated *RARRES1* as an important tumor suppressor gene by regulating versatile cellular processes like cell proliferation, differentiation, and survival [3]–[5]. For instance, RARRES1 restoration in leukemic K562 cells cooperated with all-trans retinoic acid to induce cell apoptosis [3]. Likewise, RARRES1 impeded cell proliferation and invasive features of nasopharyngeal carcinoma cells mediated by Epstein-Barr virus [4]. Furthermore, RARRES1 modulated the differentiation of subcutaneous adipose tissue-derived mesenchymal stem cell [5]. However, the tumor suppressive effect of RARRES1 on breast carcinomas has not been proven so far, but began to be illustrated in current report.

To date, promoter hypermethylation was shown to downregulate RARRES1 expression in a variety of cancers [3], [6]–[8]. In support of this notion, our group recently discovered that hypermethylation at the *RARRES1* promoter flanking sequences can be induced by an exposure to breast cancer-associated fibroblasts [9]. Yet, the past reports haven’t precisely defined the crucial cis-element responsible for epigenetic silencing of *RARRES1* by methylation nor how various DNA segments interact with each other and with transcription factors. This study, therefore, employed a high throughput technology with fine resolution, namely MassARRAY, for quantifying the levels of methylation occurring at either individual or at the clusters of CpG dinucleotides [10]. We assessed the levels of methylation at sequences flanking *RARRES1* promoter in 18 pairs of breast tumor cores plus adjacent benign tissues as well as in breast cancer cell lines and have discovered methylation at two regions can exert strikingly distinct epigenetic outcomes. While distal sequences (−664∼−86) displayed negligible promoter activity and methylation of which conferred unnoticeable silencing effect, the proximal region flanking sequences −85∼+420 harbored remarkable promoter function and methylation of which downregulated gene expression. Perhaps, methylation at the former motif preludes a methylation spread into the latter segment where epigenetic silencing effect virtually takes place.

On the other hand, CCCTC-binding factor (CTCF) is a highly conserved zinc finger protein with diverse functions involving not only transcriptional regulation, but also DNA methylation as well as organization of global chromosomal architecture through boundary/insulator formation [11]–[13]. CTCF contains a central DNA-binding domain consisting of 11 zinc fingers that confers its ability to bind to a wide range of DNA sequences [11], [12]. Inferred by data generated from chromatin immunoprecipitation (ChIP) coupled by microarray chip arrays (know as ChIP-chip), a putative CTCF binding motif was predicted in sequences flanking *RARRES1* transcriptional start site (TSS) [14] (Figure S1). We therefore investigated whether binding to CTCF was able to regulate *RARRES1* transcription experimentally. Our data revealed that CTCF occupancy was not only enriched at the unmethylated *RARRES1* promoter harboring transcription-active histone markings, but also positively correlated with RARRES1 expression such that knocking down CTCF was able to suppress *RARRES1* transcription. Together, this study redefined an important promoter element responsible for epigenetic silencing of *RARRES1* by DNA methylation and by impeding the binding to CTCF. Moreover, the roles RARRES1 plays in breast neoplasm remain largely undiscovered to date. However, this report demonstrated that *RARRES1* acts as a breast tumor suppressor in part by enhancing cell death after chemotherapeutic agent treatments and by impeding metastatic spread. Silencing of *RARRES1*by methylation and by a loss of CTCF binding possibly augmented neoplastic properties associated with advanced breast carcinomas.

## Materials and Methods

### Patients’ Specimens and Cell Lines

Fresh breast tumors and the matched adjacent benign tissues were procured from the Department of Pathology of the Ohio State University (OSU), in compliance with the institutional review board of OSU with an approved protocol number 2008C0048. Patients’ clinical information can be found in the Table S1. Immediately after procurement, tissues were macrodissected by our qualified pathologist and flash frozen at −80°C until DNA and RNA extraction.

Normal human mammary epithelial cells derived from three different women (designated as HMEC-1, -2 and -3) were purchased from Lonza and from ScienceCell Research Laboratories (Carlsbad, CA). Cells were cultured in mammary epithelial growth medium (MEGM) (Lonza). All breast cancer cell lines used in this study were generous gifts from Dr. Max S. Wicha [15]. MCF10A cells were grown in DMEM/F12 medium supplemented with 5% horse serum, Epidermal Growth Factor (EGF; 20 ng/ml), insulin (10 µg/ml), hydrocortisone (500 ng/ml), and cholera toxin (100 ng/ml). BrCa-MZ-01 and SK-BR-7 cells were proliferated in RPMI1640 plus 10% Fetal Bovine Serum (FBS) whereas SUM149 and SUM159 cells were propagated in F12 medium with 5% FBS and hydrocortisone (1 µg/ml). For inducing RARRES1 expression in SUM159 cells, tetracycline (Tet)-free FBS was employed in lieu of regular FBS. Unless specified elsewhere, the remaining cells were grown in DMEM with 10% FBS. Moreover, antibiotic-antimycotic (Invitrogen) was routinely added to all culture media for preventing microbial contamination.

### Quantification of DNA Methylation by MassARRAY Technology

To finely quantify the levels of methylation occurring at the sequences flanking *RARRES1* promoter, a high-throughput system namely MassARRAY platform (Sequenom) was utilized as described previously [9].

### Retroviral Vectors and Infections

Retroviral plasmids, pRetroX-Tet-On Advanced and pRetroX-Tight-Pur vectors, were purchased from Clontech. In principle, to generate recombinant retroviruses, plasmids were respectively transfected into packaging cells namely Phoenix™ Ampho (Orbigen, Inc.; San Diego, CA) mediated by calcium phosphate. 24 hours later, medium was replenished and the resultant supernatant, enriched with retroviruses, was collected at a 12-hour interval twice, pooled, and then stored at −80°C. For expressing RARRES1 under an inducible condition, the coding region of *RARRES1* was amplified by polymerase chain reactions (PCR) and then cloned into pRetroX-Tight-Pur vector mediated by *Bam*HI and *EcoR*I restriction cleavages (New England Biolabs). The resultant chimera (pRetroX-Tight-Pur-*RARRES1*) was affirmed to be mutation-free by DNA sequencing and then packed into recombinant retrovirus. To ectopically express RARRES1 in SUM159 cells, cells were maintained in Tet-free medium for at least one passage and then infected by pRetroX-Tet-On Advanced viruses followed by a drug selection. The G418 resistant cells were subsequently infected with retrovirus encoding for pRetroX-Tight-Pur-*RARRES1* followed by a selection using two agents: G418 (500 µg/ml) and puromycin (2.5 µg/ml).

### Downregulating CTCF by ShRNA

To knockdown CTCF in cells originally expressing high levels of RARRES1, pSM2c-based retroviral vectors encoding either scrambled or CTCF-specific shRNA (purchased from Open Biosystems, Huntsville, AL) were transfected into cells to be tested and followed by a selection with puromycin (2.5 µg/ml). Subsequent experiments investigating the effect of CTCF silencing on RARRES1 expression were carried out in cells derived from 3^rd^ passages after drug selection.

### Assessing the Effect of Methylation on Promoter Activity by in Vitro Methylation Followed by Luciferase Assays

The pGL3-Basic plasmid (Promega) reporting firefly luciferase activity was integrated with various fragments amplified from the *RARRES1* promoter flanked with *Xho*I and *Hind*III (New England Biolabs) restriction sites. The inserts in the chimera were proven to be free-of-mutation by sequencing.

*In vitro* methylation was performed as described previously [16]. Briefly, 20 µg of chimera pGL3-Basic plasmid was cleaved by *Xho*I and *Hind*III to retrieve the fragments encompassing *RARRES1* sequences. The resultant insert moiety was divided into two equal fractions and then each was either untreated (namely mock) or treated with CpG methyltransferase *Sss*I (New England Biolabs). After affirming the methylation status by a digestion with methylation-sensitive restriction enzymes *Aci*I (New England Biolabs), inserts were ligated back into the pGL3-Basic, and then introduced into 293 cells by using Lipofectamine 2000 (Invitrogen) for luciferase assays. Furthermore, to serve as a control for normalization, same cells were concordantly transfected with Renilla luciferase vector. 48 hours later, cells were lysed and luminescence was measured by Dual-Luciferase Reporter Assay kit (Promega). The diminished ratio of firefly to renilla values thus indicates a loss of promoter activity due to methylation.

### ChIP

ChIP was carried out as described previously [17]. Briefly, cells were fixed with 1% formaldehyde and then the resultant DNA-protein complexes were sonicated followed by immunoprecipitation using antibodies against H3K4me2 (Millipore), H3K27me3 (Diagenode), CTCF (Millipore) as well as the control normal rabbit IgG (Millipore). After dissociating the DNA-protein complexes, pulled-down DNA along with the input DNA (devoid of antibody) were subject to qPCR analysis using primers specifically interrogating different portions of the *RARRES1* promoter. Folds of enrichment were calculated by ratio of signals derived from ChIP DNA to those from the original input.

### Western Blot Analysis

Cells were lysed in RIPA lysis buffer (Cell signaling) supplemented with protease inhibitor cocktail tablet (Roche). Approximately 30–50 µg of proteins were resolved by 8–10% SDS-PAGE and immunoblotted with antibodies against RARRES1 (R&D Systems), CTCF (Millipore) and GAPDH (Cell Signaling) respectively.

### Invasion Assay

SUM159 variant stably transduced with pRetroX-Tight-Pur-*RARRES1* retroviruses were either treated with vehicle DMSO or with Doxycycline (500 ng/mL) for 24 hours. Single-cell mixture was obtained by trypsinization followed by suspension in the reduced-serum medium (2% FBS), in the absence or presence of Doxycycline. 1.25×10^4^ of the resultant cells were loaded to Matrigel-coated invasion chambers (8 µm pore size; BD Biosciences) to quantify the invasive property. Invasion process lasted 24 hours and was triggered by the medium that was placed outside of the invasion chamber and supplemented with 10% FBS (either without or with Doxycycline). The nonmotile cells located at the top of the filter were sweep off by cotton swabs while the motile cells transversed to the bottom of the filter were fixed with 70% ethanol and stained with 0.1% crystal violet for cell counting. The average number of migrated cells was quantified by the counts cumulated from 10 representative areas captured by a microscope with 200-fold magnification.

### Immunohistochemical (IHC) Staining

Six sets of formalin-fixed paraffin-embedded sections, each comprises primary tumor, adjacent benign, and metastatic lymph node, were obtained from the Department of Pathology in our institution. Clinical information was provided in the Table S2. IHC staining was performed using the Histostain-*Plus* kit (Invitrogen) according to the manufacturer’s instruction. Briefly, paraffin sections were deparaffinized with xylene and rehydrated in a series of descending concentrations of ethanol. Antigen retrieving was carried out by immersing the slide in sodium citrate buffer (pH 6.0) at 95°C for 15 minutes, while quenching the endogenous peroxidase activity was achieved by incubating with 3% hydrogen peroxide. Non-specific epitopes were blocked prior to incubating with specific antibody against RARRES1 (Sigma) overnight at 4°C. On the next day, sections were incubated with biotin-conjugated secondary antibody. The resultant immunocomplexes were visualized by streptavidin-conjugated enzyme along with DAB chromogen, counter-stained with hematoxylin (Invitrogen), dehydrated, preserved and then viewed under a BX45 Clinical Microscope (Olympus).

### Reverse Transcription Followed by Quantitative PCR Analysis (RT-qPCR)

Total RNA was extracted from cells by using Trizol (Invitrogen) and 1.0 µg of which served as templates for generating the complementary DNA (cDNA) mediated by SuperScript III reverse transcriptase (Invitrogen). The resultant cDNA products were mixed with RT^2^ SYBR Green qPCR Master Mixes (Qiagen) followed by quantitative PCR analysis on a 7500 fast real-time PCR machine (Applied Biosystems). Gene expression was normalized to the control transcript glyceraldehyde-3-phosphate dehydrogenase (*GAPDH*). Primer sequence used for qPCR is available in the Table S3.

### Survival Assay

SUM159 variant conditionally expressing RARRES1 was treated with mock vehicle (DMSO) or with Doxycycline (500 ng/mL) for 24 hours and then seeded in 96-well plates at a density of 5,000 per well. On the next day, cells were either treated with vehicle or with various concentrations of drugs (doxorubicin or paclitaxel) for 2 days. Viability was quantified by using MTT test (Sigma). Mock treated cells were set as 100% in relation to the cells treated with drugs.

### Statistical Analysis

The Student’s *t* test was conducted to analyze the significance of variations between the control as well as experimental samples and *p*<0.05 was regarded as significant.

## Results

### Methylation Occurring at the Region Upstream and Distal from the *RARRES1* Promoter Exerted Negligible Silencing Effect in Primary Breast Tumors

**Figure 1:**
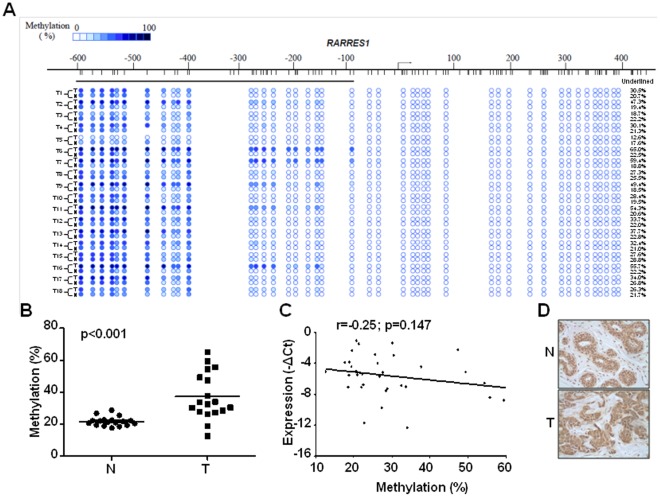
Methylation occurring at sequences upstream and distal to the *RARRES1* promoter exerted a negligible silencing effect in primary breast tumors. (A) Primary breast tumors along with their matched adjacent benign tissues (total 18 cases examined) were analyzed for DNA methylation by MassARRAY assay. Within the schema flanking *RARRES1* promoter, each vertical line represented a single CpG site, while each circle at the lower panel indicated various methylation level of a CpG unit that contained either single or multiple CpG sites. Sample names were outlined at the left, while the average methylation levels occurring at the underlined region (−664∼−86) were denoted at the right. T, tumor core; N, adjacent benign. (B) Dot plot revealed a significant gain of methylation at the underlined region in tumor cores compared to the ones in adjacent benign tissues (p<0.001). (C) Scatter plot inferred expression of RARRES1 was unrelated to level of methylation at the same underlined sequences. (D) Similar levels of RARRES1 expression between the breast tumor cores and the adjacent benign tissues were demonstrated by immunohistochemical staining. A representative image was captured from case 1 (Table S2).

To delineate how methylation at various regions flanking the *RARRES1* promoter interact with each other and with cofactors for exerting ultimate silencing effect, we began to evaluate the methylation occurring at sequences between −664 and +420 (reference to TSS set as +1) in 18 pairs of primary breast tumors as well as their matched benign tissues. In agreement with previous findings [3], we observed that many tumors displayed higher methylation levels than those obtained from the benign tissues adjacent to the tumor cores (Figure 1A) with statistical significance (*p*<0.001, Figure 1B). However, it is noteworthy that hypermethylation solely occurred at the region far upstream from the TSS (denoted as the distal region, spanning −664∼−86; underlined region in Figure 1A) while the downstream sequences (denoted as the proximal region, flanking −85∼+420) remained largely unmethylated (Figure 1A). Since promoter methylation commonly results in gene silencing, we next analyzed the correlation between the levels of methylation and the degrees of RARRES1 expression. Surprisingly enough, these two parameters were negligibly correlated inferred by *p* value as being 0.147 (Figure 1C). To substantiate this finding, IHC staining was performed to evaluate RARRES1 in 6 independent cases. In a strong agreement, both geographic sections (tumor cores and benign tissues) expressed comparable level of RARRES1 in all subjects examined (a representative case is shown in Figure 1D).

### Methylation of the Proximal Region of *RARRES1* Promoter was Necessary and Sufficient for Exerting Epigenetic Repression Effect

**Figure 2:**
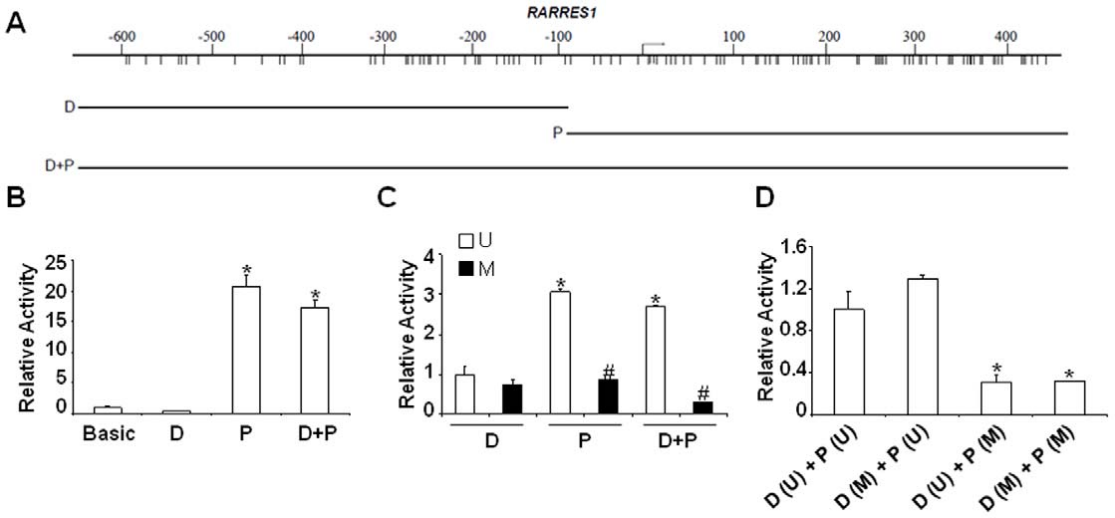
Flanking sequences proximal to the *RARRES1* promoter displayed a full promoter activity that can be suppressed by DNA methylation. (A) Fragments (D, P and D+P) flanking the *RARRES1* promoter were inserted to pGL3-Basic luciferase vector. (B) The resultant constructs were transfected into 293T cells followed by luciferase assays for assessing promoter activity. While activity from pGL3-Basic was set as 1, the one from Renilla luciferase vector served as an internal control for normalization. (C) D, P, or D+P regions were either mockly treated (Unmethylated, U) or methylated *in vitro* (M). The resultant fragments were further ligated to the luciferase vector backbone (pGL3-Basic) and subject to luciferase analysis. Significance of difference (if p<0.01) was denoted by comparing unmethylated P and D+P to that of D (marked by *) or methylated (M) to those of unmethylated counterparts (U) in samples P and D+P (denoted as #). (D) Various promoter activities were associated with swap constructs (D and P), respectively derived from either the unmethylated (U) or methylated (M) state. The full activity was obtained from unmethylated D(U)+P(U) set as 1, while * depicted a significant loss ascribed to methylation (p<0.01).

To investigate the above paradoxical finding that hypermethylation of *RARRES1* at upstream distal sequences failed to exert noticeable inhibitory effect on gene expression, we further unraveled elsewhere regions that might possess intrinsic promoter activity. As shown in Figure 2A, *RARRES1* promoter and flanking sequences were arbitrarily divided into a distal (denoted as D, −664∼−73) and proximal (labeled as P, −91∼+576) segments (Figure 2A). Interestingly enough, region P, but not D, displayed apparent promoter activity (∼20 folds higher than that of basic control) and was similar to the one from the combined region (D+P) (Figure 2B). Our data thus indicated that region P alone is sufficient to exhibit the vast majority of promoter activity. To further investigate whether region P is susceptible to silence by methylation, *in vitro* methylation assay was employed. Importantly, methylation resulted in a dramatic decline of promoter activity intrinsic to the P and D+P regions (Figure 2C). In contrast, the same treatment negligibly lowered activity associated with region D (Figure 2C). Assay reliance was inferred by the data showing that mock treated (i.e. unmethylated) P and D+P regions still displayed significantly higher promoter activity than the D region (Figure 2C). Furthermore, promoter swap test was carried out by cutting off the region linking D and P regions *via Nco*I followed by a re-ligation of exchanged fragments. It was apparent that epigenetic silencing was largely ascribed to methylation at the P region, regardless in the context of methylated or unmethylated D (first 2 columns in Figure 2D). It is noteworthy to mention that methylated D plus unmethylated P regions somewhat recapitulated the physiological mosaic at the *RARRES1* promoter observed in primary breast tumors (Figure 1A) and is in a strong agreement with a lack of silencing effect (Figures 1C and 1D).

### Methylation at the Proximal Region of *RARRES1* Promoter Rendered Silencing Effect in Breast Cancer Cell Lines

**Figure 3:**
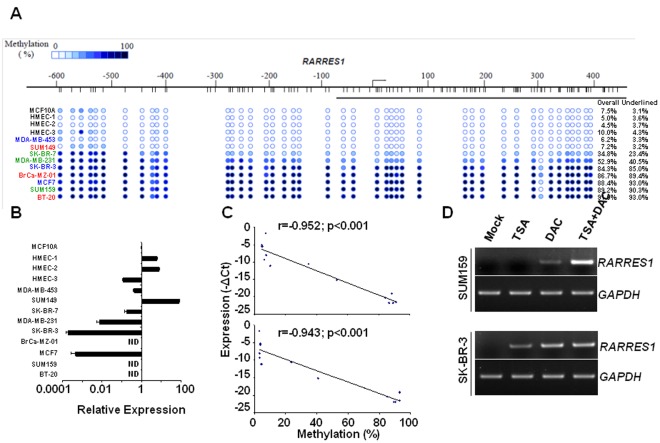
Methylation at the region proximal to the *RARRES1* promoter rendered silencing effect in breast cancer cell lines. (A) Degrees of methylation occurring at the sequence flanking the *RARRES1* promoter (D+P) in normal breast epithelia and in cancer cell lines were ranked in an ascending order. Names of breast cancer cell lines were color-coded as: blue for luminal (*N* = 3), red for basal (*N* = 3) and green for mesenchymal (*N* = 3). (B) Relative RARRES1 expression in the indicated cells was evaluated by RT-qPCR and compared to that of MCF10A set as 1. ND, too low to be detected. (C) Scatter plot depicted an inverse correlation between RARRES1 expression and methylation occurring at either overall (−664∼+420, upper panel) or merely at the proximal region (−85∼+420, lower panel) in the aforementioned cell lines. (D) Treatment of SUM149 and SK-BR-3 cells with epigenetic drugs reversed the silencing effect and re-stored expression inferred by increased transcripts assessed by RT-PCR.

In order to seek a proof that methylated region P indeed silenced RARRES1 expression, we correlated the methylation of *RARRES1* promoter with expression of transcript in not only breast cancer cell lines classified into 3 different subtypes, i.e. luminal, basal and mesenchymal [15], [18], but also in the non-neoplastic breast cell line (MCF10A) and primary normal human mammary epithelial cells derived from different women (HMEC-1, -2, and -3). As shown in Figure 3A, 5 out of 9 breast cancer cell lines, i.e. SK-BR-3, BrCa-MZ-01, MCF7, SUM159, and BT-20 exhibited dramatic methylation (>80%), regardless of origins of subtypes. SK-BR-7 and MDA-MB-231 cells showed moderate methylation (30%–60%), while MDA-MB-453 and SUM149 as well as the remaining nonmalignant breast epithelial cells displayed low methylation (<10%). Despite that various breast cancer cell lines displayed differential methylation levels at the *RARRES1* promoter (Figure 3A), they all retained similar degrees of methylation at the constitutive hypermethylated locus know as *LINE-1* (Long Interspersed Nucleotide Element 1) serving as a positive control for global methylation [19] (Figure S1). Nevertheless, the finding that all (100%) of nonmalignant breast epithelial cells harbored negligible methylation (top 4 specimens in Figure 3A) has supported our aforementioned notion that methylation at sequences flanking *RARRES1* promoter is highly associated with breast carcinomas. Notably, methylation uniformly spanned between regions D and P and displayed a pattern strikingly distinct from that of primary tumors (compare Figure 3A to Figure 1A). As cancer cell lines harboring elevated methylation were predominantly originated from metastatic or effusion sites (MCF7, MDA-MB-231, SK-BR-3 and SK-BR-7), it is reasoned that metastastatic spread is correlated with methylation in region P, despite that region D might have been methylated during the preceding non-metastatic state. In supporting of this notion, all cells (100%) displaying methylated region P concordantly harbored methylated region D, suggesting the latter incidence prelude the former. Alternatively, it might be reasonable to speculate that the discrepancy of methylation patterns between the cultured cell lines and the primary tumors might be ascribed to either an outgrowth followed by a clonal enrichment (during cell culture) of the sub-population that already acquired hypermethylated region P or an induction of hypermethylation at region P following *in vitro* cell culture. Nevertheless, the 5 cell lines harboring remarkably elevated methylation (>80%), particularly at region P, have dramatically lost *RARRES1* transcript (Figure 3B) and the reduction of expression correlated with the degrees of methylation occurring at the P region-containing segments (D+P or P) (*p*<0.001, Figure 3C). This finding agreed with the data denoting methylation at region P, but unlikely region D, is important for exerting epigenetic silencing effect (Figures 2C and 2D).

To affirm that DNA methylation indeed plays a role in silencing of *RARRES1*, SUM159 and SK-BR-3 cells were treated with epigenetic drugs (DAC or TSA or both) to partly reverse epigenetic effect. DAC alone enabled restoration of RARRES1 expression in both cell lines originally silenced by methylation, affirming that methylation was one of the critical causes rendering silencing effect (Figure 3D). Of note, DAC synergized with TSA to reactivate RARRES1 expression in SUM159 but not in SK-BR-3, indicating that dual mechanisms (methylation in conjunction with histone de-acetylation) played far critical roles in the former whereas methylation alone was sufficient to exert a silencing impact on the later cell line (Figure 3D).

### CTCF was Associated with Active, but Not Silenced, *RARRES1* Promoter and thereby Regulated its Expression

**Figure 4:**
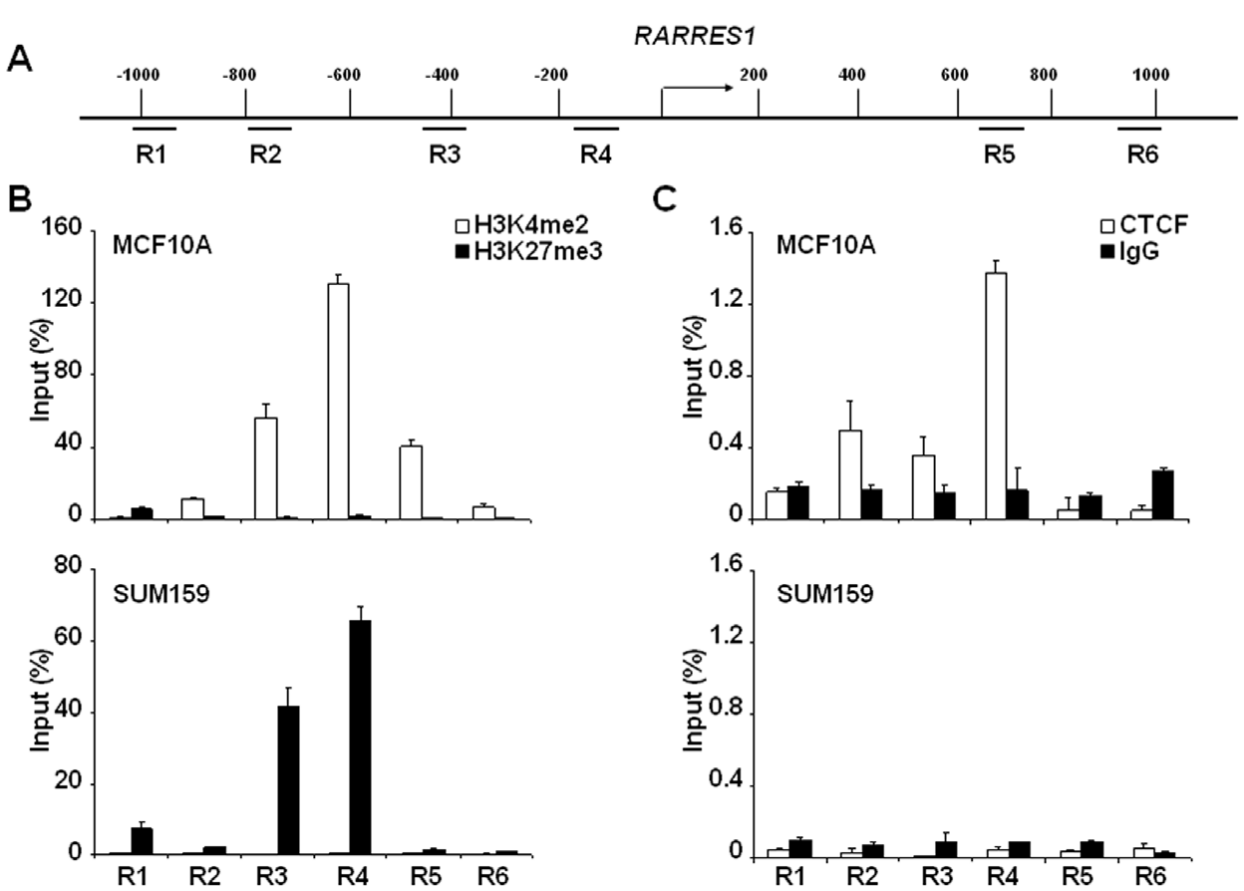
*RARRES1* promoter was poised by active histone marking (H3K4me2) and co-occupied with CTCF in the expressing cells (MCF10A), as opposed to the occupancy of repressive histone H3K27me3 with depleted CTCF binding in the non-expressing cells (SUM159). (A) A schematic map revealed *RARRES1* promoter as well as its flanking sequences (denoted as R1∼R6) that were interrogated by ChIP-qPCR assay. (B) RARRES1-expressing MCF10A and -silenced SUM159 cells were subjected to ChIP-qPCR analysis for assessing the abundance of histone modifications. (C) CTCF occupancy was enriched in regions R4 in MCF10A but was absent in SUM159. Quality reliance is assured by a negligible binding to normal IgG.

Recently, multifunctional protein CTCF was reported to epigenetically regulate expression of various tumor suppressors including *p16*, *BRCA1*, *Rb*, *PUMA* and *p53*, possibly through insulating chromatin boundaries such that prevented the spread of upstream repressive chromatin [20]–[24]. ChIP-chip assay implicated a putative CTCF binding motif existed in sequences flanking −83 bp to −39 bp upstream of TSS of *RARRES1* [14] that displayed sequence conservation with the one identified in the *H19* promoter [25] (Figure S2A). We further reasoned that CTCF occupancy perhaps acts as an insulator by prohibiting methylation to further invade into region P and subsequently prevented epigenetic silencing. Under this notion, binding to CTCF may be influenced by surrounding histone markings. Herein, ChIP-qPCR was carried out to analyze the occupancy of histone molecules histone 3 lysine 4 dimethylation (H3K4me2) and histone 3 lysine 27 trimethylation (H3K27me3), two common histone variants respectively indicative of transcriptionally-active and -repressed chromatins [26]. In RARRES1-expressing (MCF10A) and -silenced (SUM159) cells, we observed significantly elevated occupancy of H3K4me2 in the former whereas increased association of H3K27me3 in the latter, suggesting that a pronounced chromatin remodeling has occurred in cells situated at opposing expression states (Figure 4B). Interestingly, occupancy of CTCF was predominantly correlated with coexistence of H3K4me2 at the same promoter region (R4, a region flanking sequences −154∼−73 bp upstream of TSS) in MCF10A cells, suggesting that this region might comprise a part of the “core” promoter element mediated by binding to CTCF and to H3K4me2 (upper panels in Figure 4B and 4C). In support of this notion, binding of CTCF and H3K4me2 at the *RARRES1* promoter was similarly observed in RARRES1-expressing SUM149 but not in silenced SK-BR-3 cells (data not shown). As ChIP-qPCR indicated that the enrichment of CTCF binding peaked at the R4 (Figure 4C), we theorize this region might be involved in blocking methylation spread into region P exemplified in the primary tumors (Figure 1A). This issue might be delineated in the future studies, by assessing the effect of mutated the CTCF core binding motif (flanking the *RARRES1* promoter) on abrogating the “insulating” effect.

**Figure 5:**
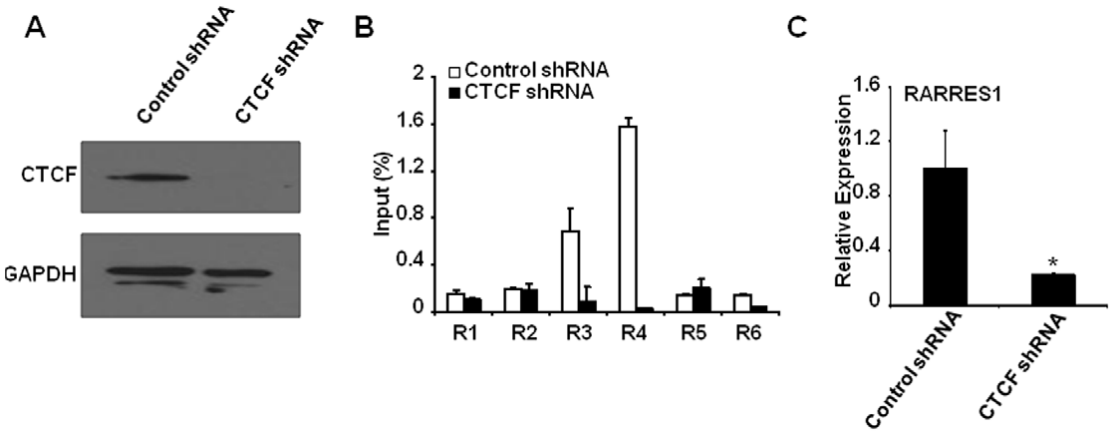
Knocking down CTCF resulted in a significant loss of RARRES1 expression. (A) MCF10A cells were transduced with recombinant viruses to express either control or CTCF shRNA, followed by a Western blot analysis to capture the expression of CTCF (upper) or GAPDH (lower, served as a loading control). (B) Same cells were subjected to ChIP-qPCR analysis for assessing CTCF occupancy at regions R1-R6. (C) Knocking down CTCF resulted in silencing of RARRES1 that can be inferred by RT-qPCR analysis. Significant loss of transcript was denoted by * if p<0.01.

To further prove that the binding of CTCF played a key role in regulating RARRES1 expression, shRNA was implemented to down-regulate CTCF in MCF10A cells (Figure 5A). Interestingly, knocking down CTCF not only resulted in a loss of its binding to *RARRES1* promoter (Figure 5B), but also reduced RARRES1 expression by >5-fold (Figure 5C). Taken together, our data supported that binding of CTCF to the *RARRES1* promoter is important for sustaining the promoter at the transcription-prone state.

### Re-expression of RARRES1 Impeded Cell Invasion and Promoted Apoptosis

**Figure 6:**
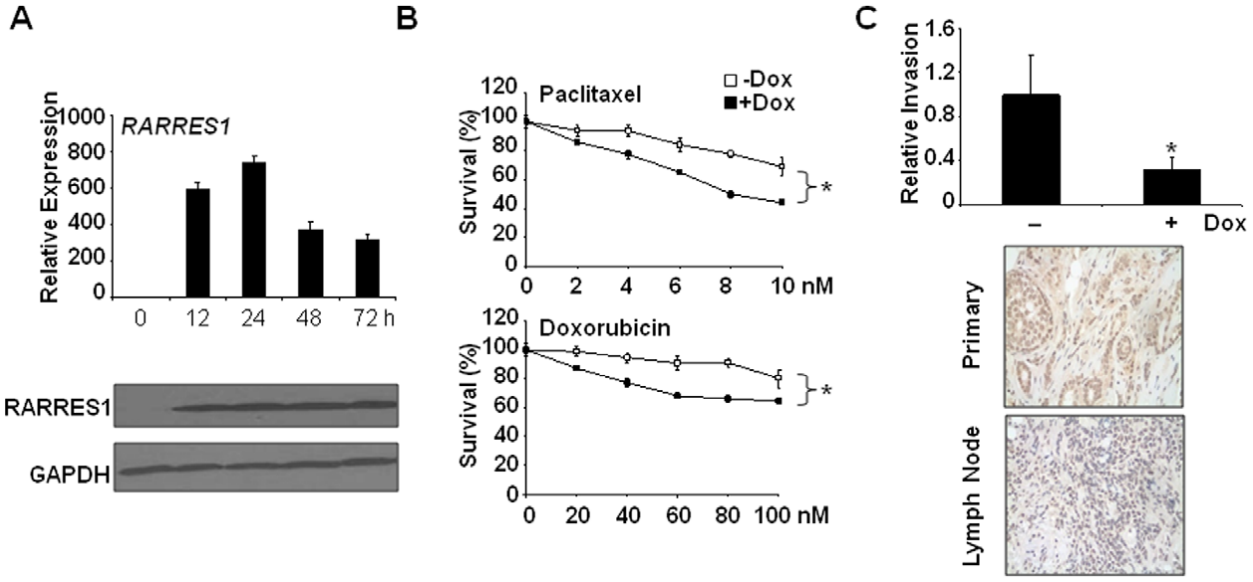
RARRES1 restoration impeded invasion and promoted cell death. (A) Re-expression of RARRES1 was induced in SUM159 variants after been incubated with Doxycycline (Dox) for various durations. Expression level was assessed by RT-qPCR (upper) and by Western blot analysis (lower), in comparison with an internal control GAPDH. (B) The same RARRES1-expressing SUM159 variant was either untreated or treated with Dox for 24 hours. The resultant cells were then challenged with various doses of Paclitaxel and Doxorubicin for two days and the survival cells were quantified by MTT assays. * inferred a significant change of cell viability (p<0.05). (C) Upper, a significant loss of invasive ability occurred in SUM159 variant re-expressing RARRES1. *, p<0.05. Lower, representative IHC images indicated a reduced RARRES1 expression in metastatic lymph nodes compared to primary breast tumors of the same patient (cases 2 shown in Tables S2 and S4).

To investigate how silenced RARRES1 contributed to breast neoplastic phenotypes, we engineered a Tet-On system in SUM159 cells in which RARRES1 expression was originally silenced but can be later induced upon the addition of doxycycline. As shown in Figure 6A, both mRNA and protein levels of RARRES1 increased drastically at 12 hours and peaked at 24 hours after the addition of doxycycline (Figure 6A). In concurrence with previous reports examining various human cancers [2]–[4], [6]–[8], [27], we observed that re-expression of RARRES1 not only augmented cell death induced by cytotoxic agents Paclitaxel and Doxorubicin (Figure 6B), but also impeded cell invasion (upper panel of Figure 6C).

To gain clinical insight regarding how RARRES1 silencing influences the dissemination of malignant cells, expression was quantified between primary breast tumors and their corresponding metastatic lymph nodes (total 6 cases examined, Tables S2 and S4) by employing IHC staining using a RARRES1-specific antibody. In agreement with our data that re-expression of RARRES1 inhibited cell invasion (upper panel, Figure 6C), RARRES1 was generally down-regulated in metastatic lymph nodes compared to those in the primary tumors (lower panel of Figure 6C) in all cases examined (Table S4). Though metastasis property correlated with a gain of tumor-initiating cells enriched in the ALDEFLUOR-positive subfraction [28]–[31], RARRES1-restoration didn’t render a loss of breast cancer-initiating cells (data not shown), excluding the likelihood that RARRES1 regulates this activity. Taken together, our data supported *RARRES1* to be a tumor suppressor gene in breast cancer and its downregulation, by DNA methylation, CTCF binding, or by histone remodeling, might favor dissemination and survival of breast carcinoma. However, our current data cannot delineate the chronological sequences between these perturbations.

## Discussion

Field cancerization was recently theorized [32], although aberrant epigenetic silencing by methylation *via* a step-wise manner hasn’t been extensively exemplified nor been correlated with the cancer progression. For the first time, our preceding report [9], as well as current study, depicted a likelihood that tumor suppressor locus, *RARRES1*, was progressively methylated prior to undergoing epigenetic silencing. Subsequently, downregulated *RARRES1* triggered various malignant properties and was associated with advanced neoplastic states in various cancer types [2]–[4], [6]–[8], [27] and in breast carcinomas (current report).

The nonmalignant breast epithelia MCF10A cells initially harbored negligible methylation at sequences flanking *RARRES1* promoter (Figure 3A), but later gained remarkable methylation occurring at region D (or perhaps region P as well) after an exposure to the tumor microenvironment provoked from breast cancer-associated fibroblasts [9]. Gain of methylation was demonstrated to be related to geographic distance that is inferred from our data obtained from primary tumor cores versus the matched adjacent benign tissues (Figure 1A) and is consistent with the methylation spread theory [32], [33]. Surprisingly enough, the suppressing effect was not prominent until methylation was extended to the neighboring element: region P (Figure 3A-C).

Despite that the upstream factors rendering aberrant methylation of region D was likely the tumor-environment *via* contacting cancer-associated fibroblasts [9], the etiologic cause(s) rendering region P methylation remains largely unknown. Presumably, methylation at the region D acts as a catalyst to (A) recruit the methylation machinery or histone modifications or (B) interact between the two, or (C) synergize with additional effector molecules like CTCF and H3K27me3. Notably, continuous presence of methylated region D might be important for expanding methylation into region P and leading to ultimate silencing. The methylation profiles of regions D and P from the non-malignant breast epithelial cells and from cancer cell lines studied in this report revealed a “none-and-all” pattern but not an “either-or” fashion (Figure 3A).

Our data suggested a notion that “methylation seed” perhaps existed in region D. This phenomenon agreed with published findings that scattered CpG sites within a CpG island originally acted as “catalysts” without necessarily exerting noticeable silencing effect under normal circumstances, but later gained methylation when cells progressed to malignant states. For example, in gastric cancer cell lines, “methylated seeds” were abnormally elevated that subsequently augmented methylation of CpG island prior to conducting a permanent repression of downstream genes [34], [35]. Likewise, triggered by downregulated *SP1* transcription factor, random seeds of methylation acted as a catalyst for the spread of methylation across the CpG island of the Glutathione *S*-transferase promoter [36]. The seeds of DNA methylation can also trigger histone deacetylation followed by histone methylation, denoting a temporal relationship between gene expression, DNA hypermethylation, and chromatin remodeling in cancer cells, particularly in prostate carcinomas [33].

The spread of methylation from the “seed” to the adjacent sequences was worthwhile to mention. Methylation spread across biparental origins was manifested in patients with Prader–Willi syndrome-like features displaying hypo-pigmentation symptoms. The maternal X-chromosome was not only inactivated by methylation, but its aberrant methylation was also furthered into the paternal chromosome 15 leading to the abnormal hypermethylation and silencing of downstream targets *SNRPN* and *OCA2* [37]. Similarly, selective “seed” methylation occurring at the large tandem repeats becomes important for the subsequent extension of the critically methylated region that resulted in stable silencing of a locus namely *FWA* and thus prevented late flowering in *Arabidopsis thaliana* [38]. On the other hand, demethylation of both CpG and non-CpG methylation can be similarly expanded and thus re-activate the *myogenin* transcript during muscle differentiation [39], suggesting an epigenetic mechanism by “spreading” stretches of methylcytosines might be a common occurrence in both CpG and non-CpG context.

Alternative mechanisms other than methylation “seed” are worthy to be speculated. Instead of exerting direct impact, methylated region D might indirectly interplay with other co-factors or transcription factors or CpG-island shores that subsequently augment local hypermethylation at region P. In mouse methylome, CTCF binding was shown to be necessary and sufficient to create a low-methylated regions and this effect was partly ascribed to localized demethylation function associated with CTCF [13]. In our study, by serving as a cis-acting element, methlayted region D perhaps abrogated the occupancy of CTCF at *RARRES1* promoter (R4 region, −154∼−72 upstream of TSS, Figures 4A and 4C) and thus impaired CTCF’s “insulation” effect as well as loss of de-methylation followed by a again of methylation in the neighboring region P. Alternatively, methylated region D might convey aberrant methylation at un-identified distal CpG island shores [40] and this perturbation may subsequently augment methylation of P-region, perhaps *via* a chromosome-looping mechanism [41], [42]. It is noteworthy to point out that the influence of aberrantly methylated CpG island shores on epigenetic silencing was recently denoted in human carcinomas. By comparing colon tumors with patient-matched normal tissue, Feinberg laboratory identified cancer-associated differentially methylated regions were not only involved in transcriptional silencing but were also located at CpG island shores rather than the classical CpG islands or promoters [43].

Since CTCF appeared to be important for sustaining *RARRES1* promoter at a transcription-prone state, it is of interest to speculate how CTCF might become dysfunctional in RARRES1–silenced cells. First of all, posttranslational modifications such as poly(ADP-ribosyl)ation (PARylation) of CTCF might be involved in regulating transcription activities as well as influencing DNA methylation of target genes [20], [44], [45]. However, in current study, CTCF-180 (highly PARylated) and CTCF-130 (scantly PARylated) isoforms [44] were similarly shown in RARRES1-expressing (MCF10A) and –silenced (SUM159) cell lines, despite that CTCF-180 was negligibly detected in both cell lines (Figure S3). This finding excluded the likelihood that PARylation of CTCF plays a key role influencing the binding to *RARRES1* promoter in our study. Alternatively, aberrant DNA methylation occurring at region D might abrogate CTCF binding to its cognate sequence within region P, as exemplified in the control of imprinted *Igfr2/H19* loci [25].

Thus far, our findings cannot clearly evolve the mechanistic sequence deciphering how CTCF binding influences methylation and regulates expression of RARRES1. During the progression of breast carcinomas, perhaps, the non-malignant stage would sustain region D at the unmethylated state and thus facilitate the binding of CTCF to R4 region of *RARRES1* promoter by which methylation at region P can be prevented. Later carcinogenic insults or exposure to breast cancer-associated fibroblasts [9] might augment hypermethylation at region D and thus impaired its binding to transcription-prone histone markings, cofactors, as well as to CTCF. Perhaps, loss of CTCF occupancy abrogates demethylation activities intrinsically associated with CTCF and thereby leads to methylation spread moving towards region P. This perturbation thus results in silencing of RARRES1 expression seen in the metastatic cultured cell lines (Figure 3C) as well as in the primary lymph nodes manifesting the advanced stages of breast neoplasm (Figure 6C).

Not only step-wise methylation flanking *RARRES1* promoter depicted a proof-of-principle, but also how it’s silencing impacts malignant phenotypes is of great interest. Five lines tested in this report (MCF7, MDA-MB-231, SK-BR-3, and SK-BR-7) harboring silenced RARRES1 were virtually isolated from metastatic or effusion sites, substantiating the notion that RARRES1 is a negative regulator for invasion and metastasis and that its silencing was a common perturbation associated with lymph node metastasis (Figure 6C). However, exceptional cases in cell lines harboring prominent metastatic features but retained full *RARRES1* transcript might be ascribed to additional undiscovered perturbations (other than RARRES1 suppression) that are also important for augmenting invasion and promoting metastasis properties.

Furthermore, the outcome ascribed to RARRES1 silencing coincided with its clinical applications for treating human cancers. For example, in conjunction with imatinib, all-trans retinoid acid was used to treat gastrointestinal stromal tumors by impeding cell proliferation and inducing apoptosis mediated through down-regulated survivin as well as up-regulated Bax protein [46]. Moreover, the *in vitro* and *in vivo* effects of retinoids either alone or in combination with cisplatin and 5-fluorouracil on tumor development and metastasis of melanoma were assessed. Retinoids not only showed significant anti-proliferation and anti-invasion effects on murine melanoma B16-F10 cells, but also augmented the antitumor activity of cisplatin *in vivo* [47].

Collectively, our findings delineated multiple molecular perturbations are responsible for epigenetic silencing of RARRES1, in the light of the tumor microenvironmental effect, DNA methylation, CTCF binding, as well as histone modifications. Identifying the etiologic factor leading to methylation expansion (into region P) is apparently important for developing therapeutic strategies. Future treatment regimen by abrogating this trigger and thus blocking region P methylation followed by sustaining RARRES1 expression could potentially improve disease prognosis *via* hampering metastasis, the common cause of death in a wide range of human carcinomas including breast cancer.

## Supporting Information

Figure S1 **All breast cancer cell lines examined in this report retained hypermethylation at the constitutive methylated locus named** ***LINE-1*** **.** To provide a methylation control across cell lines studied in this report, we further investigated the methylation degree of a globally methylated locus know as *LINE-1* (Long Interspersed Nucleotide Element 1) [1] by a semi-quantitative assay known as combined bisulfite restriction analysis (COBRA) [2]. DNA was extracted from the respective cell lines and treated with sodium bisulfite followed by a PCR amplification using the primers that do not contain CpG dinucleotides so that the amplification step would not be influenced by their original methylation status. The amplified products were further subjected to restriction digestions to discern methylated from the unmethylated DNA of interest. Briefly, the combination of bisulfite treatment and PCR amplification results in the sustenance of methylated cytosines thereby retains the susceptible to *BstU*I digestion (inferred from the production of restricted fragments). Under the same treatment, unmethylated cytosines are converted to thymines and thus become resistant to *BstU*I cleavage (denoted by a lack of restriction fragment). The DNA template used in the positive control (denoted as Meth) was the CpGenome Universal Methylated DNA (Millipore #S7821). For generating a negative control (labeled as Un-Meth), the same template was subjected to an extra step of PCR amplification prior to bisulfite conversion such that methylated moieties can be erased. Nevertheless, COBRA assay revealed that all cell lines retained similar magnitudes of methylation at *LINE-1* promoter, disregard differential methylation degrees have occurred at *RARRES-1* promoter.
1. Belancio VP, Roy-Engel AM, Pochampally RR, Deininger P (2010) Somatic expression of LINE-1 elements in human tissues. Nucleic Acids Res 38: 3909–3922. 2. Xiong Z, Laird PW (1997) COBRA: a sensitive and quantitative DNA methylation assay. Nucleic Acids Res 25: 2532–2534.
(PPTX)

Figure S2 **The putative CTCF binding motif flanking** ***RARRES1*** **promoter displays sequence homology with the one in the B1 region of** ***H19*** **. A.** The potential CTCF binding motif located between −83 bp and −39 bp upstream of transcriptional start site (TSS) of *RARRES1* [3] was compared to the *H19* B1 CTCF binding segment [4]. Conserved bases were revealed by the MAFFT method as previously described [5] and shown in gray shades. **B.** Scheme of binding motifs flanking the promoter region −200 bp upstream of *RARRES1* TSS. Boxes denote locations of potential CTCF binding sites evolved from our current study (denoted as Peng *et al*.) as well as from a previous report [3] (labeled as Kim *et al*.). The various transcription factor binding sites [6] flanking *RARRES1* promoter were illustrated by an on-line software http://www.cbrc.jp/research/db/TFSEARCH.htm. Abbreviations for the transcription factors are: **Sp**1: Specificity Protein 1; **GATA1**: GATA binding factor 1; **GATA1/2**, GATA binding factor 1 and GATA binding factor 2; **E2F:** Adenoviral E2 promoter binding factor; and **MZF1**: myeloid zinc finger 1.
3. Kim TH, Abdullaev ZK, Smith AD, Ching KA, Loukinov DI, et al. (2007) Analysis of the vertebrate insulator protein CTCF-binding sites in the human genome. Cell 128: 1231–1245.4. Hark AT, Schoenherr CJ, Katz DJ, Ingram RS, Levorse JM, et al. (2000) CTCF mediates methylation-sensitive enhancer-blocking activity at the H19/Igf2 locus. Nature 405: 486–489.5. Katoh K, Misawa K, Kuma K, Miyata T (2002) MAFFT: a novel method for rapid multiple sequence alignment based on fast Fourier transform. Nucleic Acids Res 30: 3059–3066.6. Heinemeyer T, Wingender E, Reuter I, Hermjakob H, Kel AE, et al. (1998) Databases on transcriptional regulation: TRANSFAC, TRRD and COMPEL. Nucleic Acids Res 26: 362–367.
(PPT)

Figure S3 **CTCF isolated from RARRES1-expressing (MCF10A) and –silenced cells (SUM159) displayed similar degree of poly(ADP-ribosyl)ation (PARylation).** Detection of PARylation (denoted as PAR) by mmunoprecipitation (IP) followed by western blotting was performed as previously described [7]. In brief, semi-confluent culture of MCF10A or SUM159 cells grown on 10-cm dishes were lyzed in 1000 ?l of IP buffer (Pierce IP kit #26146) supplemented with protease inhibitor cocktail (Roche). The lysate were incubated for 16 hours with either anti-PAR-10H mouse monoclonal antibody (Enzo Life Technology # ALX-804-220) or with anti-PAR rabbit polyclonal antibody (EMD Millipore #528815) and then incubated respectively with Protein A/G Agarose (Pierce #20422) or with HRP-Protein A Agarose bead (BD#610438) for 2 hours. Immunoprecipitated products were analyzed by western blotting using anti-CTCF polyclonal antibodies (Millipore #07-729). As both RARRES1-expressing (MCF10A) and –silenced (SUM159) cells harbor CTCF with similar levels of poly(ADP-ribosyl)ation (PARylation), it is unlikely that a loss of CTCF binding in the latter cell lines (Figure 4C) was ascribed to a decreased PARylation.
7. Farrar D, Rai S, Chernukhin I, Jagodic M, Ito Y, et al. (2010) Mutational analysis of the poly(ADP-ribosyl)ation sites of the transcription factor CTCF provides an insight into the mechanism of its regulation by poly(ADP-ribosyl)ation. Mol Cell Biol 30: 1199–1216.
(PPTX)

Table S1 Clinicopathological information of breast tumors assessed for DNA methylation by MassARRAY analysis. DCIS, ductal carcinoma in situ; IDC, invasive ductal carcinoma; ILC, invasive lobular carcinoma; LCIS, lobular carcinoma in situ.
(DOCX)

Table S2 Clinicopathological information of breast tissues for IHC staining.
(DOCX)

Table S3 DNA sequences of primers used in this study.
(DOCX)

Table S4 Summary of RARRES1 IHC (immunohistochemical) staining.
(DOCX)
