# Supplementary figures and images for: Epigenetic Repression of RARRES1 Is Mediated by Methylation of a Proximal Promoter and a Loss of CTCF Binding
Source: PLoS One. 2012 May 17;7(5):e36891. doi: 10.1371/journal.pone.0036891 (PMC3355180; doi:10.1371/journal.pone.0036891)

## Slide 1
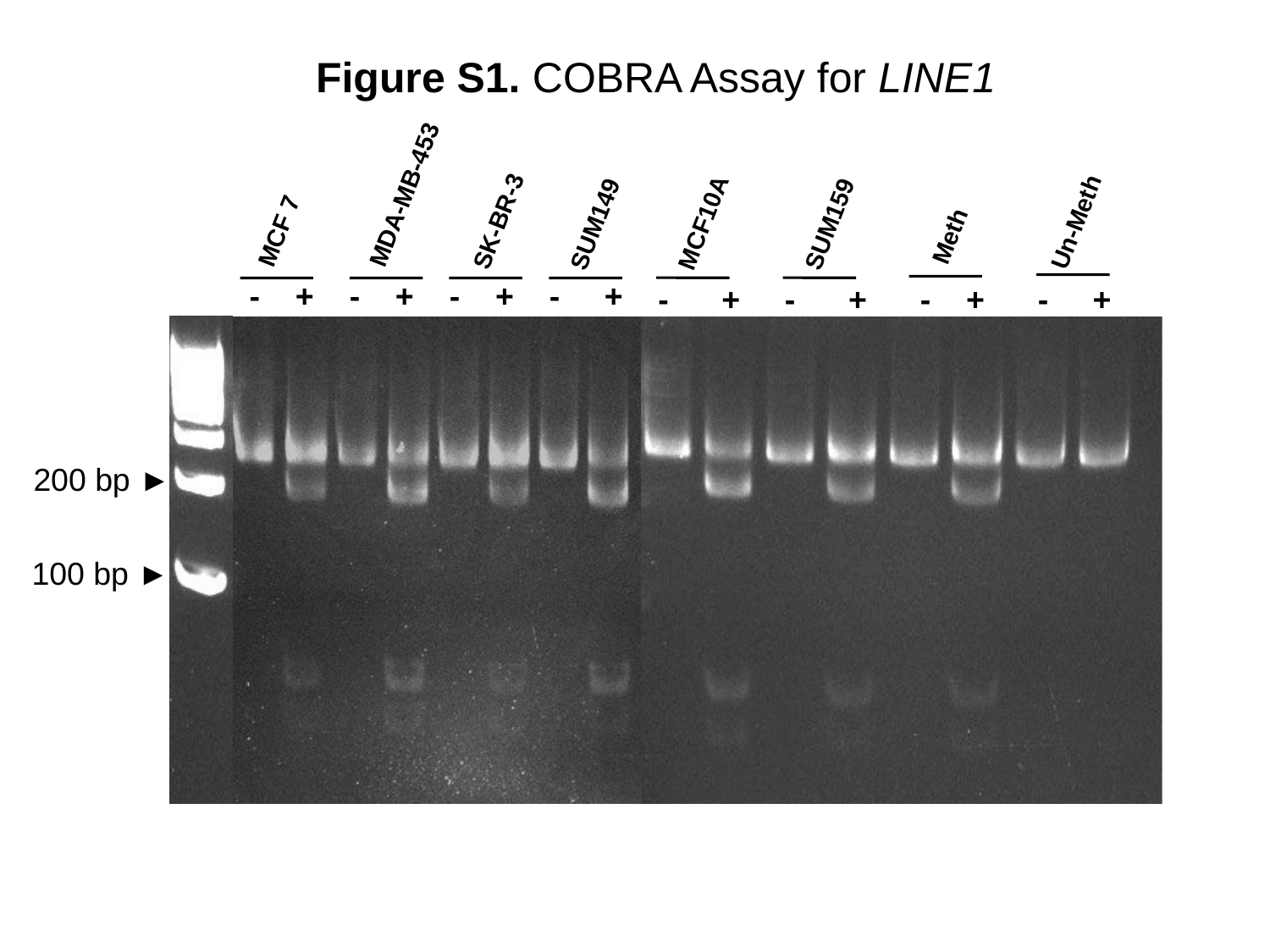

Figure S1. COBRA Assay for LINE1
MDA-MB-453
SK-BR-3
Un-Meth
MCF10A
SUM159
SUM149
MCF 7
Meth
 - + - + - + - +
- + - + - + - +
200 bp ►
100 bp ►

Supplement: Figure S1 — All breast cancer cell lines examined in this report retained hypermethylation at the constitutive methylated locus named LINE-1 . To provide a methylation control across cell lines studied in this report, we further investigated the methylation degree of a globally methylated locus know as LINE-1 (Long Interspersed Nucleotide Element 1) [1] by a semi-quantitative assay known as combined bisulfite restriction analysis (COBRA) [2]. DNA was extracted from the respective cell lines and treated with sodium bisulfite followed by a PCR amplification using the primers that do not contain CpG dinucleotides so that the amplification step would not be influenced by their original methylation status. The amplified products were further subjected to restriction digestions to discern methylated from the unmethylated DNA of interest. Briefly, the combination of bisulfite treatment and PCR amplification results in the sustenance of methylated cytosines thereby retains the susceptible to BstUI digestion (inferred from the production of restricted fragments). Under the same treatment, unmethylated cytosines are converted to thymines and thus become resistant to BstUI cleavage (denoted by a lack of restriction fragment). The DNA template used in the positive control (denoted as Meth) was the CpGenome Universal Methylated DNA (Millipore #S7821). For generating a negative control (labeled as Un-Meth), the same template was subjected to an extra step of PCR amplification prior to bisulfite conversion such that methylated moieties can be erased. Nevertheless, COBRA assay revealed that all cell lines retained similar magnitudes of methylation at LINE-1 promoter, disregard differential methylation degrees have occurred at RARRES-1 promoter. 1. Belancio VP, Roy-Engel AM, Pochampally RR, Deininger P (2010) Somatic expression of LINE-1 elements in human tissues. Nucleic Acids Res 38: 3909–3922. 2. Xiong Z, Laird PW (1997) COBRA: a sensitive and quantitative DNA methylation assay. Nucleic Acid [file pone.0036891.s001.pptx]
