# Supplementary material for: Epigenetic Repression of RARRES1 Is Mediated by Methylation of a Proximal Promoter and a Loss of CTCF Binding
Source: PLoS One. 2012 May 17;7(5):e36891. doi: 10.1371/journal.pone.0036891 (PMC3355180; doi:10.1371/journal.pone.0036891)
Supplement: Figure S3 — CTCF isolated from RARRES1-expressing (MCF10A) and –silenced cells (SUM159) displayed similar degree of poly(ADP-ribosyl)ation (PARylation). Detection of PARylation (denoted as PAR) by mmunoprecipitation (IP) followed by western blotting was performed as previously described [7]. In brief, semi-confluent culture of MCF10A or SUM159 cells grown on 10-cm dishes were lyzed in 1000 ?l of IP buffer (Pierce IP kit #26146) supplemented with protease inhibitor cocktail (Roche). The lysate were incubated for 16 hours with either anti-PAR-10H mouse monoclonal antibody (Enzo Life Technology # ALX-804-220) or with anti-PAR rabbit polyclonal antibody (EMD Millipore #528815) and then incubated respectively with Protein A/G Agarose (Pierce #20422) or with HRP-Protein A Agarose bead (BD#610438) for 2 hours. Immunoprecipitated products were analyzed by western blotting using anti-CTCF polyclonal antibodies (Millipore #07-729). As both RARRES1-expressing (MCF10A) and –silenced (SUM159) cells harbor CTCF with similar levels of poly(ADP-ribosyl)ation (PARylation), it is unlikely that a loss of CTCF binding in the latter cell lines (Figure 4C) was ascribed to a decreased PARylation. 7. Farrar D, Rai S, Chernukhin I, Jagodic M, Ito Y, et al. (2010) Mutational analysis of the poly(ADP-ribosyl)ation sites of the transcription factor CTCF provides an insight into the mechanism of its regulation by poly(ADP-ribosyl)ation. Mol Cell Biol 30: 1199–1216. (PPTX) [file pone.0036891.s003.pptx]

## Slide 1
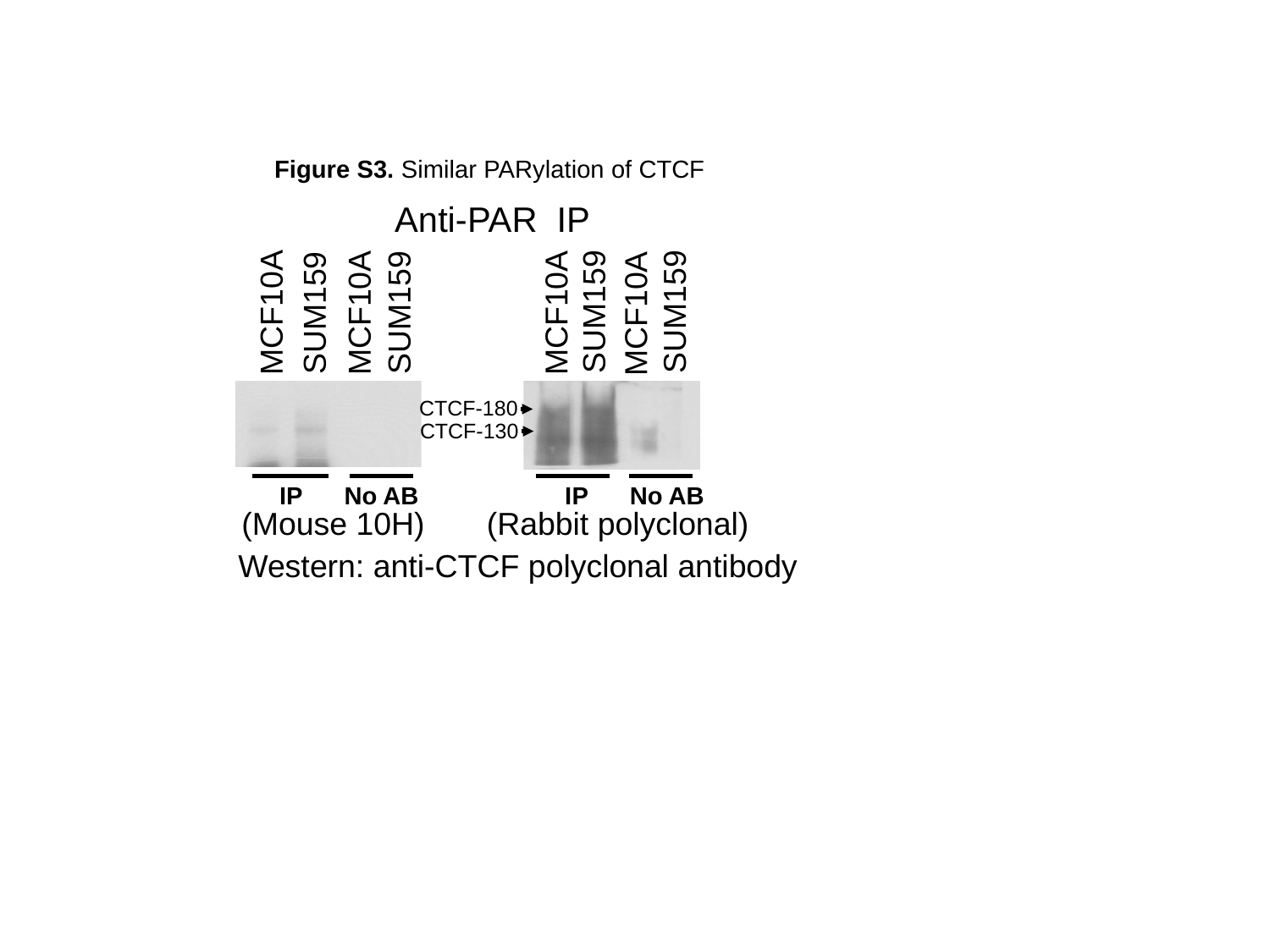

Figure S3. Similar PARylation of CTCF
Anti-PAR IP
SUM159
SUM159
MCF10A
MCF10A
SUM159
SUM159
MCF10A
MCF10A
CTCF-180
CTCF-130
 IP No AB IP No AB
(Mouse 10H) (Rabbit polyclonal)
Western: anti-CTCF polyclonal antibody
