# Supplementary material for: Epigenetic Repression of RARRES1 Is Mediated by Methylation of a Proximal Promoter and a Loss of CTCF Binding
Source: PLoS One. 2012 May 17;7(5):e36891. doi: 10.1371/journal.pone.0036891 (PMC3355180; doi:10.1371/journal.pone.0036891)
Supplement: Table S1 — Clinicopathological information of breast tumors assessed for DNA methylation by MassARRAY analysis. DCIS, ductal carcinoma in situ; IDC, invasive ductal carcinoma; ILC, invasive lobular carcinoma; LCIS, lobular carcinoma in situ. (DOCX) [file pone.0036891.s004.docx]

| **Table S1. Clinicopathological Information of Breast Tumors Assessed for DNA Methylation by MassARRAY Analysis.** | | | | | | | | |
| --- | --- | --- | --- | --- | --- | --- | --- | --- |
| Case | Age/Race | Histology diagnosis | Tumor stage (TNM ) | Grade (Bloom-Richardson) | Lymph metast (met./total) | ER | PR | HER2 |
| T1 | 62/white | ILC, LCIS | IIB (pT2 N1a Mx) | 1, well differentiated | Yes (1/27) | Positive | Positive | Negative |
| T2 | 28/white | IDC, DCIS | IIIA (pT2 N2 Mx) | 3, poorly differentiated | Yes (7/27) | Negative | Negative | Positive |
| T3 | 51/white | ILC, LCIS | IIA (pT2 N0 Mx) | 2, moderately differentiated | No (0/3) | Positive | Positive | Positive |
| T4 | 43/white | ILC, LCIS | IIA (pT2 N0 Mx) | 1, well differentiated | Yes (1/3) | Positive | Positive | Negative |
| T5 | 39/white | ILC, LCIS | IIIB (pT4a N1a Mx) | 2, moderately differentiated | Yes (3/5) | Positive | Negative | Positive |
| T6 | 68/white | IDC, DCIS | IIA (pT2 N0 Mx) | 1, well differentiated | Yes (1/5) | Positive | Positive | Negative |
| T7 | 72/white | IDC | IIA (pT2 N0 Mx) | 2, moderately differentiated | No (0/12) | Positive | Positive | Negative |
| T8 | 63/black | IDC, DCIS | IIB (pT2 N1 Mx) | 3, poorly differentiated | Yes (1/26) | Negative | Negative | Negative |
| T9 | 79/white | IDC | IIA (pT2 N0 Mx) | 3, poorly differentiated | No (0/2) | Positive | Positive | Negative |
| T10 | 62/white | IDC, DCIS | IIA (pT1c N1 Mx) | 2, moderately differentiated | Yes (3/17) | Positive | Positive | Negative |
| T11 | 62/white | IDC, DCIS | IIIB (pT4b N3a Mx) | 3, poorly differentiated | Yes (13/42) | Positive | Negative | Negative |
| T12 | 64/white | IDC, DCIS | IIB (pT2 N1 Mx) | 3, poorly differentiated | Yes (1/13) | Negative | Negative | Negative |
| T13 | 35/black | IDC | IIIA (pT3 N1 Mx) | 1, well differentiated | Yes (1/6) | Positive | Positive | Negative |
| T14 | 64/white | IDC, DCIS | IIIA (pT2 N2a Mx) | 3, poorly differentiated | Yes (7/23) | Positive | Positive | Positive |
| T15 | 34/white | IDC, DCIS | I (pT1a N0) | 2, moderately differentiated | Yes (10/20) | Negative | Negative | Positive |
| T16 | 87/white | IDC, DCIS | IIIB (pT4b N3a Mx) | 3, poorly differentiated | Yes (36/36) | Positive | Negative | Positive |
| T17 | 68/white | IDC, ILC, DCIS, LCIS | IIA (pT2 N0) | 1, well differentiated | No (0/10) | Positive | Positive | Negative |
| T18 | 64/white | IDC, DCIS | IIIA (pT3 N1 Mx) | 3, poorly differentiated | Yes (2/17) | Negative | Positive | Negative |
|  | | | | | | | | |
