# Supplementary material for: Epigenetic Repression of RARRES1 Is Mediated by Methylation of a Proximal Promoter and a Loss of CTCF Binding
Source: PLoS One. 2012 May 17;7(5):e36891. doi: 10.1371/journal.pone.0036891 (PMC3355180; doi:10.1371/journal.pone.0036891)
Supplement: Table S2 — Clinicopathological information of breast tissues for IHC staining. (DOCX) [file pone.0036891.s005.docx]

| **Table S2. Clinicopathological Information of Breast Tissues for IHC staining** | | | | | | | | |
| --- | --- | --- | --- | --- | --- | --- | --- | --- |
| Case | Age | Histology diagnosis | Tumor stage (TNM ) | Grade (Bloom-Richardson) | Lymph metast (met./total) | ER | PR | HER2 |
| 1 | 51 | IDC, DCIS | IIIC (pT2 N3a Mx) | 3, poorly differentiated | Yes (12/39) | Positive | Positive | Negative |
| 2 | 44 | IDC, DCIS | IIIC (pT1c N3a) | 1, well differentiated | Yes (10/23) | Positive | Positive | Negative |
| 3 | 57 | ILC, LCIS | IIIB (pT3 N3a Mx) | 1, well differentiated | Yes (31/43) | Positive | Positive | Negative |
| 4 | 72 | IDC, DCIS | IIIA (pT2 N2a Mx) | 3, poorly differentiated | Yes (4/31) | NA | NA | NA |
| 5 | 65 | IDC, DCIS | IIB (pT2 N1 Mx) | 3, poorly differentiated | Yes (1/26) | Negative | Negative | Negative |
| 6 | 30 | IDC, DCIS | IIIA (pT2 N2 Mx) | 3, poorly differentiated | Yes (7/27) | Negative | Negative | Positive |
|  | | | | | | | | |
