# Supplementary material for: Epigenetic Repression of RARRES1 Is Mediated by Methylation of a Proximal Promoter and a Loss of CTCF Binding
Source: PLoS One. 2012 May 17;7(5):e36891. doi: 10.1371/journal.pone.0036891 (PMC3355180; doi:10.1371/journal.pone.0036891)
Supplement: Table S3 — DNA sequences of primers used in this study. (DOCX) [file pone.0036891.s006.docx]

| **Table S3. DNA Sequences of primers used in this Study** | | |
| --- | --- | --- |
|  |  | Primer sequences 5' to 3' |
| *RARRES1* MassARRAY-1 | Forward | AGGAAGAGAGTTTTTAGTTTAGTTTAGGGATTTG |
|  | Reverse | CAGTAATACGACTCACTATAGGGAGAAGGCTTTACTACCCACTCCTCCCTC |
| *RARRES1* MassARRAY-2 | Forward | AGGAAGAGAGGTAGTTTTTGGGTTTTTTTTGT |
|  | Reverse | CAGTAATACGACTCACTATAGGGAGAAGGCTCCTACTAAAAAATCACAACCCT |
| *RARRES1* MassARRAY-3 | Forward | AGGAAGAGAGGGGATTTTAGTATTTTGAGGTT |
|  | Reverse | CAGTAATACGACTCACTATAGGGAGAAGGCTACAAAAAAAACCCAAAAACTAC |
| *RARRES1* MassARRAY-4 | Forward | AGGAAGAGAGGGTAGTTTTAGGATGTTGGGG |
|  | Reverse | CAGTAATACGACTCACTATAGGGAGAAGGCTTACCCAAATATCACCTCCCAAC |
| *RARRES1* expression vector | Forward | TTAGGATCCATGCAGCCCCGCCGGCAAC |
|  | Reverse | TTAGAATTCTTAGAAATTACTAAGCTCTGTTGG |
| *RARRES1* promoter (-664/-73) luciferase | Forward | TAACTCGAGGCTTCTGAGACAAATGGGA |
|  | Reverse | TAAAAGCTTCACTCCTTTTCCACGTTTC |
| *RARRES1* promoter (-91/+576) luciferase | Forward | TAACTCGAGGAAACGTGGAAAAGGAGTG |
|  | Reverse | TAAAAGCTTAGGAACGAGCCAGATTTC |
| *RARRES1* promoter (-664/+576) luciferase | Forward | TAACTCGAGGCTTCTGAGACAAATGGGA |
|  | Reverse | TAAAAGCTTAGGAACGAGCCAGATTTC |
| *RARRES1 ChIP_R1* | Forward | TGCCCGGCTAATTTTTGTAT |
|  | Reverse | GCTCACGAGGTCAGGAGTTT |
| *RARRES1 ChIP_R2* | Forward | CACTGTGCGAGGCAGATTTA |
|  | Reverse | AACACTTGCTGCCTCCATTC |
| *RARRES1 ChIP_R3* | Forward | CCAAGCATTAGGGCTGTGAT |
|  | Reverse | GACTTCTCCCACCTCCACAG |
| *RARRES1 ChIP_R4* | Forward | CACTCCTTTTCCACGTTTCC |
|  | Reverse | ATGCCGCATCCTAGCACTAA |
| *RARRES1 ChIP_R5* | Forward | AGTAAGCACCCCCATAGCAA |
|  | Reverse | TTGGGAAATTGAGGAACAGG |
| *RARRES1 ChIP_R6* | Forward | ATGCCTTGAAATCAGCAACC |
|  | Reverse | GCTGGCGTCTGATTTGATCT |
| *RARRES1 RT-qPCR* | Forward | ACGGCTCATCGAGAAAAAGA |
|  | Reverse | GAAAGCCAAATCCCAGATGA |
| *GAPDH RT-qPCR* | Forward | CCCCTTCATTGACCTCAACTACAT |
|  | Reverse | CACTCCTGGAAGATGGTGA |
|  |  |  |
