# Supplementary material for: Epigenetic Repression of RARRES1 Is Mediated by Methylation of a Proximal Promoter and a Loss of CTCF Binding
Source: PLoS One. 2012 May 17;7(5):e36891. doi: 10.1371/journal.pone.0036891 (PMC3355180; doi:10.1371/journal.pone.0036891)
Supplement: Table S4 — Summary of RARRES1 IHC (immunohistochemical) staining. (DOCX) [file pone.0036891.s007.docx]

| **Table S4. Summary of RARRES1 IHC (immunohistochemical) staining** | | | |
| --- | --- | --- | --- |
|  | RARRES1 expression | | |
| Case | Primary tumor |  | Lymph node |
| 1 | Strong |  | moderate and weak |
| 2 | Strong |  | weak |
| 3 | Strong |  | weak |
| 4 | Strong |  | weak |
| 5 | Strong |  | weak |
| 6 | Strong |  | moderate |
